# CXCR7/ACKR3-targeting ligands interfere with X7 HIV-1 and HIV-2 entry and replication in human host cells

**DOI:** 10.1016/j.heliyon.2018.e00557

**Published:** 2018-03-01

**Authors:** Thomas D'huys, Sandra Claes, Tom Van Loy, Dominique Schols

**Affiliations:** Laboratory of Virology and Chemotherapy, Department of Microbiology and Immunology, Rega Institute for Medical Research, KU Leuven, Leuven, Belgium

**Keywords:** Infectious disease, Virology, Cell biology

## Abstract

Chemokine receptors CCR5 and CXCR4 are considered the main coreceptors for initial HIV infection, replication and transmission, and subsequent AIDS progression. Over the years, other chemokine receptors, belonging to the family of G protein-coupled receptors, have also been identified as candidate coreceptors for HIV entry into human host cells. Amongst them, CXCR7, also known as atypical chemokine receptor 3 (ACKR3), was suggested as a coreceptor candidate capable of facilitating both HIV-1 and HIV-2 entry *in vitro*. In this study, a cellular infection model was established to further decipher the role of CXCR7 as an HIV coreceptor. Using this model, CXCR7-mediated viral entry was demonstrated for several clinical HIV isolates as well as laboratory strains. Of interest, the X4-tropic HIV-1 HE strain showed rapid adaptation towards CXCR7-mediated infection after continuous passaging on CD4- and CXCR7-expressing cells. Furthermore, we uncovered anti-CXCR7 monoclonal antibodies, small molecule CXCR7 inhibitors and the natural CXCR7 chemokine ligands as potent inhibitors of CXCR7 receptor-mediated HIV entry and replication. Even though the clinical relevance of CXCR7-mediated HIV infection remains poorly understood, our data suggest that divergent HIV-1 and HIV-2 strains can quickly adapt their coreceptor usage depending on the cellular environment, which warrants further investigation.

## 1 Introduction

Human immunodeficiency virus (HIV) is the causal agent of the disease known as acquired immune deficiency syndrome (AIDS). HIV/AIDS was discovered 35 years ago and by now up to 40 million people worldwide are living with this immunosuppressive disease with still around 2 million new HIV infections every year [1, 2].

The viral entry of HIV-1 and HIV-2 occurs via the initial attachment of the HIV envelope glycoprotein (gp120) subunits to CD4 molecules on the host cell surface, thereby exposing the coreceptor binding site. Subsequently, the CD4-activated gp120 subunits typically interact with either one of the two chemokine receptors, CXC Chemokine Receptor 4 (CXCR4) or CC Chemokine Receptor 5 (CCR5), both primary coreceptors in the HIV entry process. HIV strains that infect host cells using the CCR5 coreceptor are known as M-tropic, non-syncytium-inducing, CCR5-tropic (R5) viruses, while infections via the CXCR4 coreceptor occur with T-tropic, syncytium-inducing, CXCR4-tropic (X4) HIV strains. Furthermore, there are HIV strains that can bind both CCR5 and CXCR4, the so-called dual-tropic strains. R5 strains are predominant early on in HIV infection, whereas X4 strains are linked with later disease progression [3, 4, 5, 6, 7]. Several reports have now indicated an increase of HIV-1 strains that predominantly use CXCR4 as a coreceptor for virus attachment and entry. These X4 HIV-1 strains are associated with rapid disease progression towards AIDS in infected individuals. In addition, a faster clinical course is also well-documented for individuals infected with dual-tropic (most likely X4) HIV-1 strains [8, 9, 10].

Over the years, multiple other chemokine receptors, aside from CCR5 and CXCR4, were uncovered as alternative coreceptors in the HIV entry process, albeit with varying potencies. Examples of chemokine receptors also serving as HIV coreceptors are CCR3, CX3CR1, CXCR3, CXCR5, CXCR6 and CXCR7 [11, 12, 13, 14, 15, 16, 17]. The latter chemokine receptor CXCR7, also called Atypical Chemokine Receptor 3 (ACKR3), is the focus of this study. Importantly, CXCR7 and CXCR4 share CXCL12 as their natural chemokine ligand. In addition, the chemokine CXCL11 is also able to bind and activate CXCR7 [18, 19].

Anti-CXCR7 monoclonal antibody (mAb) clones 8F11-M16 and 11G8 have proven to be effective in preventing binding of the natural CXCR7 chemokine ligands CXCL11 and CXCL12 to their cognate receptor [19, 20]. The small molecules CCX771 and VUF11207 were described to interfere with CXCL12-CXCR7 binding and to induce subsequent CXCR7 internalization [20, 21]. The small cyclic peptide TC14012, initially discovered as a highly potent CXCR4 antagonist, was also shown to block CXCL12 binding to CXCR7 and to induce CXCR7 internalization, although with lower potency [22, 23, 24, 25]. Also, the natural chemokine ligands of CXCR4 and CCR5 have been shown to specifically inhibit HIV entry [26, 27, 28, 29].

**Fig. 1:**
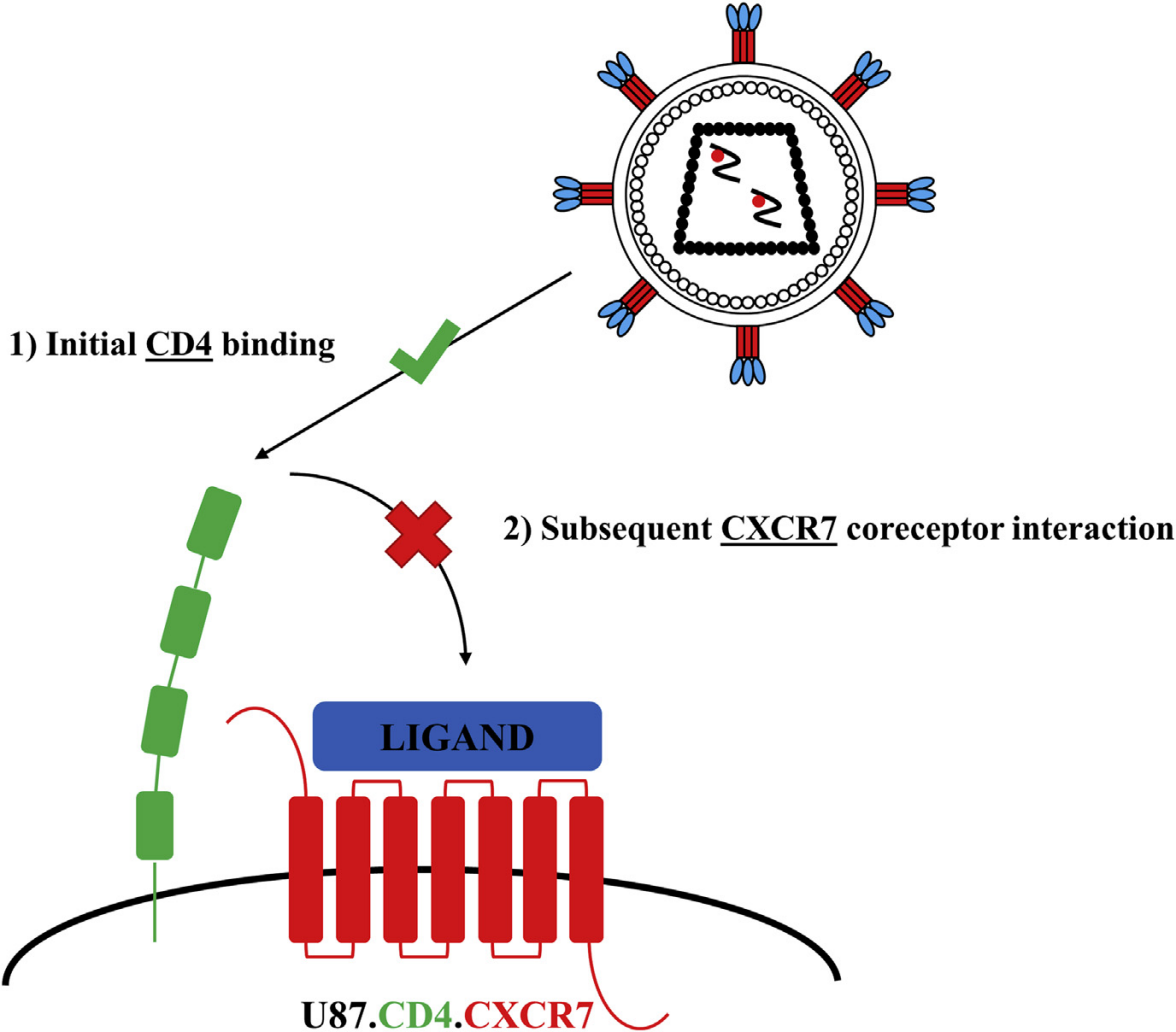
Schematic representation of an *in vitro* HIV infection model using U87.CD4.CXCR7 cells for the identification of CXCR7 coreceptor-specific inhibitors.

The chemokine receptors CXCR7 and CXCR4 not only share their natural ligand CXCL12, they also tend to heterodimerize in cells when co-expressed [18, 30]. These two findings suggest a possible role for CXCR7 as an alternative coreceptor for HIV strains, for instance due to the adaptation of CXCR4-using viruses over time, ultimately enabling them to use CXCR7 as an additional coreceptor. Therefore, in this study, we evaluated the role of CXCR7 as an alternative HIV coreceptor and investigated to what extent CXCR4-using HIV strains could use CXCR7 as coreceptor. To this end, an *in vitro* CXCR7-specific cellular infection model using human glioblastoma cells (U87-MG) was first established. Further, we evaluated whether X4-tropic strains could shift towards CXCR7 coreceptor use over time. To ultimately confirm the role of CXCR7 as an HIV coreceptor, CXCR7-targeting monoclonal antibodies (mAbs) (clones 8F11-M16, 10D1-J16 and 11G8), small molecule CXCR7 inhibitors (CCX771, VUF11207 and TC14012) and the natural CXCR7 chemokine ligands (CXCL11 and CXCL12) were applied in the infection model to demonstrate specific CXCR7 receptor-mediated infection. This also allowed to directly compare the antiviral potency of these three different classes of compounds. Fig. 1 illustrates how CD4- and CXCR7-expressing U87 cells were utilized to reveal the role of CXCR7 as an alternative HIV coreceptor and to uncover the potential inhibitory effect of different classes of CXCR7-targeting ligands on viral entry and replication of X7 HIV-1 and HIV-2 strains.

## 2 Materials and methods

### 2.1 Cell cultures, plasmids and HIV strains

The human glioblastoma U87 cell line expressing human CD4 (U87.CD4) was kindly provided by Dr. D.R. Littman (Skirball Institute of Biomolecular Medicine, New York, NY, USA). U87-MG cells without CD4 were obtained from the American Type Culture Collection (Rockville, MD, USA). HIV-1 HE strain (subtype B) was initially isolated from a Belgian AIDS patient in 1987 [31]. The primary clinical isolate NPO3 classified as HIV-1 subtype CRF01 AE was kindly provided by Dr. J. Lathey (then at BBI Biotech Research Laboratories, Gaithersburg, MD, USA). The HIV-2 EHO isolate was isolated from peripheral blood lymphocytes of a patient from Ivory Coast with full-blown AIDS [32].

### 2.2 Monoclonal antibodies, small molecules and chemokines

CXCR7-specific monoclonal antibodies (mAbs), clones 8F11-M16 and 10D1-J16 were obtained from BioLegend (San Diego, CA, USA) and clone 11G8 was obtained from R&D Systems (Minneapolis, MN, USA). ChemoCentryx (Mountain View, CA, USA) kindly provided the small molecule CXCR7 inhibitor CCX771. The CXCR7 ligands VUF11207 and TC14012 were obtained from Tocris Bioscience (Bristol, UK). CXCL11 and CXCL12, the natural CXCR7 chemokine ligands, were purchased from PeproTech (Rocky Hill, NJ, USA).

### 2.3 Stable transfection of U87-MG and U87.CD4 cells with CXCR7 wildtype

Briefly, the pTEJ-8 expression vectors encoding wild-type CXCR7, kindly provided by Thue W. Schwartz (University of Copenhagen, Denmark), were cotransfected with the pPUR selection vector encoding puromycin resistance (Clontech Laboratories, Palo Alto, CA, USA) into U87.CD4 or U87-MG cells by the use of FuGENE HD transfection reagent (Promega, USA). After puromycin (2 μg ml^−1^) selection, CXCR7-expressing cells were isolated from the puromycin-resistant cell cultures by incubation of the cells with mouse anti-human CXCR7 mAb clone 8F11-M16 (BioLegend, San Diego, CA, USA) and subsequent magnetic separation of chemokine receptor-positive cells with sheep anti-mouse immunoglobulin G (IgG)-conjugated M450 Dynabeads (ThermoFisher Scientific, Waltham, MA, USA). The transfected cells were cultured under selection in Dulbecco's modified Eagle's medium (DMEM; ThermoFisher Scientific) containing 10% fetal bovine serum (FBS; ThermoFisher Scientific), 0.01 M HEPES buffer (ThermoFisher Scientific), 0.2 mg ml^−1^ Geneticin (G-418 sulfate; ThermoFisher Scientific), and 1 μg ml^−1^ puromycin (Sigma-Aldrich, St. Louis, MO, USA).

### 2.4 Flow cytometry-based cell surface receptor staining with fluorescently labeled mAbs

U87 cells were first digested using 0.25% trypsin/EDTA and resuspended in culture medium (DMEM supplemented with 10% FBS and 1% HEPES), followed by a 2 h incubation period to allow for reappearance of receptor expression on the cell surface. U87 cells were then washed once with phosphate-buffered saline (PBS; ThermoFisher Scientific) containing 2% FBS. Cells were incubated for 30 min on ice with PE-conjugated anti-CXCR7 mAb clone 8F11-M16 (BioLegend, San Diego, CA, USA), PE-conjugated anti-CD4 mAb clone SK3 (Biolegend, San Diego, CA, USA) or PE-labeled anti-CXCR4 mAb clone 12G5 (BD Biosciences, San Diego, CA, USA) in PBS containing 2% FBS. The negative controls used in the staining experiments were the following isotype controls from BD Biosciences, mouse IgG1 (clone MOPC-21), mouse IgG2a (clone G155-178) and mouse IgG2b (clone 555743). Thereafter, the cells were washed twice with PBS, fixed in 1% paraformaldehyde (PFA) in PBS and analyzed on a FACSCalibur flow cytometer (BD, San Jose, CA, USA) in combination with FlowJo software.

For the competition experiment between unlabeled and labeled anti-CXCR7 mAbs, U87.CD4.CXCR7 cells, first digested with 0.25% trypsin/EDTA, were washed once with PBS/2% FBS. Then cells were resuspended at 4 × 10^6^ cells ml^−1^ in PBS/2% FBS, wherefrom 50 μl was used for the incubation with 10 μg ml^−1^ of the unlabeled mAb in a U-shaped 96-well plate. Cells were washed after a 30 min incubation period and incubated thereafter with the titrated dose of the labeled form of the same mAb clone. After a second incubation period, cells were washed twice and fixated in 200 μl PBS/1% PFA. The resulting samples were analyzed on a FACSCanto flow cytometer equipped with FACSDIVA software (BD, San Jose, CA, USA) in combination with FlowJo software.

### 2.5 HIV infection of CXCR7-transfected U87.CD4 cells

U87.CD4 cells transfected with CXCR7 were digested using 0.25% trypsin/EDTA. These cells were washed and resuspended at 2 × 10^4^ cells ml^−1^ in culture medium and seeded out in 24-well plates. HIV-1 laboratory strain HE and HIV-2 EHO were added at a final concentration of 5,000 pg per well p24 antigen (Ag), whereas HIV-1 NPO3 was added at 20,000 pg per well to result in proper infection. To examine the activity of CXCR7-specific mAbs and small molecules, the cells were seeded out in 24-well plates already containing the mAbs at varying concentrations (1/2 dilutions). Then, viruses were added after a preincubation period of 20 minutes and the plates were maintained at 37 °C, 5% CO_2_. The cytopathic effect (syncytium or giant cell formation) in the virus-infected cell cultures was evaluated light microscopically at 5 days after infection. Then, the supernatant was collected and analyzed for virus content based on the p24 core Ag ELISA (PerkinElmer, Zaventem, Belgium). For HIV-2 p27 Ag detection, the INNOTEST (Fujirebio, Temse, Belgium) was used. Negative control U87 cells were included expressing no (U87-MG) or only one HIV entry receptor (U87.CD4 and U87.CXCR7).

Passaging of HIV-1 HE to generate an adapted HIV-1 strain for coreceptor use was performed as follows. U87.CD4.CXCR7 cells were infected with the HIV-1 HE strain and cell cultures were incubated at 37 °C until an extensive cytopathic effect (CPE) was observed (5 days). The culture supernatant was used for further passage of HE strain virus in U87.CD4.CXCR7 cells to finally obtain an HIV-1 HE virus that was better adapted to CXCR7 (and CD4) use.

### 2.6 Flow cytometry for intracellular p24/p27 Ag detection

Five days post infection, U87.CD4.CXCR7 cells were carefully washed with cold PBS to remove excess virus and were detached by using 0.25% trypsin-EDTA for 5 min followed by cell resuspension. The percentage of HIV-infected U87 cells was determined by intracellular staining for HIV p24/p27 Ag, using the phycoerythrin (PE)-conjugated anti-p24/p27 mAb KC57-PE (Beckman Coulter, Fullerton, California, USA). Cells were analyzed on a FACSCalibur flow cytometer (BD, San Jose, CA, USA) in combination with FlowJo software.

### 2.7 HIV-1 entry PCR for visualization of early reverse transcription

We used an HIV entry PCR protocol adapted from Princen et al. [33]. U87.CD4.CXCR7 cells were seeded in 24-well plates at 10^5^ cells per well. Virus stocks (diluted to a p24 titer of 100,000 pg ml^−1^) were treated with 10 U ml^−1^ of RNase-free DNase (Roche Molecular Biochemicals, Basel, Switzerland) for 1 h at room temperature. Compound preincubation was done for 15 min. Then the cells in each well were infected with 5,000 pg of HIV-1 HE #10 p24. After incubation at 37 °C for 2 h, the cells were washed twice with PBS and total DNA was extracted from the infected cells by using the QIAamp DNA mini kit (Qiagen, Hilden, Germany) according to the manufacturer's protocol. The DNA was eluted from the QIAamp spin columns in a final volume of 50 μl elution buffer. Then 10 μl of each DNA sample was subjected to 35 cycles of HIV-1 long terminal (LTR) R/U5-specific and 30 cycles of beta-actin-specific PCR on an Eppendorf Mastercycler Nexus GX2. Each cycle comprised a 10-s denaturation step at 98 °C, a 30-s annealing step at 66 °C (HIV-1 LTR R/U5) or 72 °C (beta-actin), and a 20-s extension step at 72 °C. The primers used were as follows: LTR R/U5 sense primer, 5′-GGCTAACTAGGGAACCCACTG-3′ (nucleotides 496 to 516, according to the HIV-1 HXB-2 DNA sequence; see reference [34]); LTR R/U5 antisense primer, 5′-CTGCTAGAGATTTTCCACACTGAC-3′ (nucleotides 612 to 635); beta-actin sense primer, 5′-TCTGGCGGCACCACCATGTACC-3′ (nucleotides 2658 to 2679); beta-actin anti-sense primer, 5′-CGATGGAGGGGCCGGACTCG-3′ (nucleotides 2961 to 2980). The reaction mixtures contained PCR buffer (supplied with the enzyme), 200 μM dNTP mix (New England Biolabs, Ipswich, MA, USA), 0.5 μM (each) forward and reverse primers, and 0.5 U of Q5 Hot Start High-Fidelity DNA polymerase (New England Biolabs, USA) in a total volume of 25 μl. After gel electrophoresis through a 2.2% agarose gel (Lonza, Basel, Switzerland), we visualized the amplified DNA fragments on a FastGene FAS V Gel Documentation System (Nippon Genetics, Japan).

### 2.8 Quantitative real-time PCR for detection of virus infection and replication

Virus infection and replication in the U87.CD4.CXCR7 cell line was detected by a highly sensitive HIV-specific quantitative PCR (qPCR). U87.CD4.CXCR7 cells were seeded in 24-well microtiter plates at 25,000 cells per well and preincubated with compounds for 15 min. Virus stocks (diluted to a p24 titer of 100,000 pg ml^−1^) were treated with 10 U ml^−1^ of RNase-free DNase (Roche Molecular Biochemicals, Basel, Switzerland) for 1 h at room temperature. The cells in each well were then infected with 5,000 or 10,000 pg of HIV-1 HE #10 p24. After incubation of the HIV-infected U87.CD4.CXCR7 cell cultures at 37 °C for 4 days, the medium was aspirated, the cells were washed once with PBS, and total DNA was extracted from the infected cells with a QIAamp DNA Mini Kit (QIAGEN, Hilden, Germany). The DNA was eluted from the QIAamp spin columns in a final volume of 50 μl of elution buffer. DNA samples were analyzed by quantitative real-time PCR with an ABI Prism 7500 fast real-time PCR system (Applied Biosystems, Foster City, CA, USA) in a final volume of 20 μl containing TaqMan Fast Universal PCR Master Mix (2X), no AmpErase UNG (Applied Biosystems, USA), 900 nM each forward and reverse primer, TaqMan probe at 250 nM, and 5 μl of template DNA. The PCR cycling conditions were 20 s of initial denaturation at 95 °C, followed by 40 thermal cycles of denaturation for 3 s at 95 °C and annealing or extension for 30 s at 60 °C. Primers and probes were designed with PrimerQuest (Integrated DNA Technologies, IDT). A 86-bp fragment of the HIV-1 long terminal repeat was amplified with primers 5′-CTAGGGAACCCACTGCTTAAG-3′ (forward) and 5′-TTACCAGAGTCACACAACAGAC-3′ (reverse) (IDT, Leuven, Belgium) and TaqMan probe 5′-/FAM/CCTCAATAA/ZEN/AGCTTGCCTTGAGTGCTTCAA-/IBFQ/-3′ with double quenching (IDT, Leuven, Belgium). The same amplicon cloned into the pIDTSmart vector was bought from IDT to obtain a standard plasmid for quantifying the HIV copy number in DNA samples from infected cell cultures. In each PCR experiment, a standard curve was established with a 1:10 dilution series of known amounts of the corresponding (HIV-1) standard plasmid, starting from 10^6^ HIV-1 copies. These standard curves were used to convert the respective cycle threshold (Ct) values obtained for the DNA samples into the number of HIV proviral DNA copies.

## 3 Results and discussion

### 3.1 Stable expression of the chemokine receptor CXCR7 in U87 glioblastoma cells as determined with specific anti-CXCR7 mAbs

In order to study CXCR7-dependent biological processes, it is important to use a cell line that uniquely and stably expresses this receptor on its cell surface. Here, the human glioblastoma cell line U87-MG, an adherent cell type that does not endogenously express CXCR7, CXCR4 nor CD4 on its cellular surface, was used to generate CXCR7-expressing cell lines. The cell lines created for this study were [1] U87-MG cells expressing both the main HIV entry receptor CD4 and the chemokine receptor CXCR7 (U87.CD4.CXCR7) and [2] U87-MG cells only expressing the coreceptor CXCR7 (U87.CXCR7). The U87.CD4.CXCR7 cells were derived from previously established U87.CD4 cells, whereas U87.CXCR7 cells originated from U87-MG cells. Both U87.CD4 and U87-MG cells were stably transfected with CXCR7 cDNA contained in the pTEJ-8 mammalian expression vector.

**Fig. 2:**
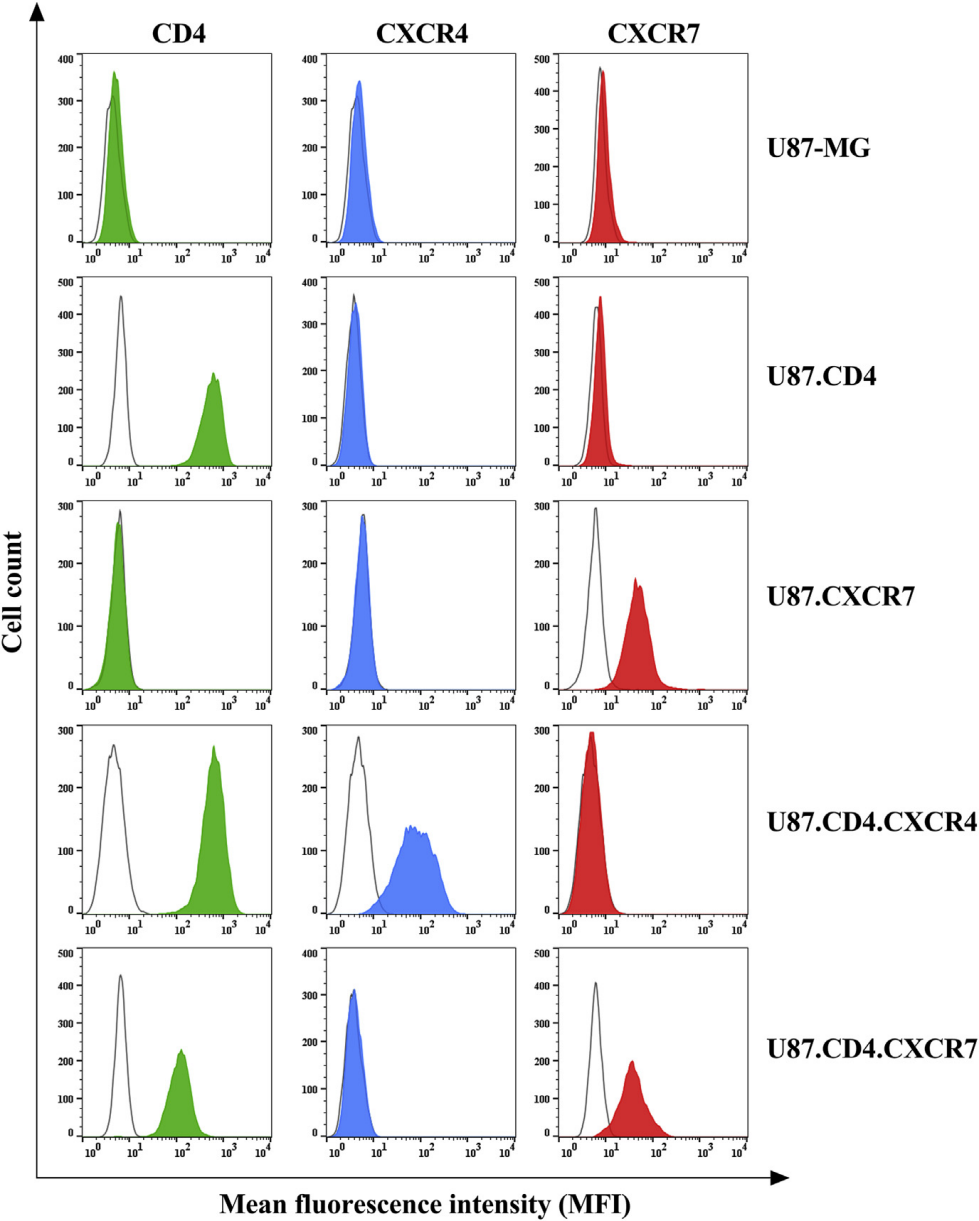
Cellular receptor staining on U87 cells determined using flow cytometry. U87-MG and U87.CD4 cells did not express chemokine receptors CXCR4 (blue) and CXCR7 (red). We show the expression of CXCR7 on the U87.CXCR7 and U87.CD4.CXCR7 cells, examined using labeled anti-CXCR7 mAb staining (red) compared to isotype control antibody staining (empty histogram). A control staining was also performed on U87.CD4.CXCR4 to check for staining selectivity of the labeled anti-CXCR4 mAb clone 12G5 (blue). CD4 expression was only detected on cell lines U87.CD4, U87.CD4.CXCR4 and U87.CD4.CXCR7 (green).

The expression level of CD4, CXCR4 and CXCR7 was demonstrated on the different U87 cell lines (Fig. 2). In order to validate protein expression of the three cell surface receptors, fluorescent PE-labeled mAbs were used that specifically bind to the target membrane receptor. Flow cytometric analysis of the antibody labeling was performed and demonstrated the purity of the original cell lines U87-MG and U87.CD4 as well as the efficient stable insertion of CXCR7 cDNA into both cell lines (Fig. 2). For cell surface staining of CXCR7, the 8F11-M16 mAb clone was used. As a positive control for the functionality of the PE-labeled anti-CXCR4 mAb clone 12G5, CXCR4 cell surface receptor staining was performed on U87.CD4.CXCR4 cells.

**Fig. 3:**
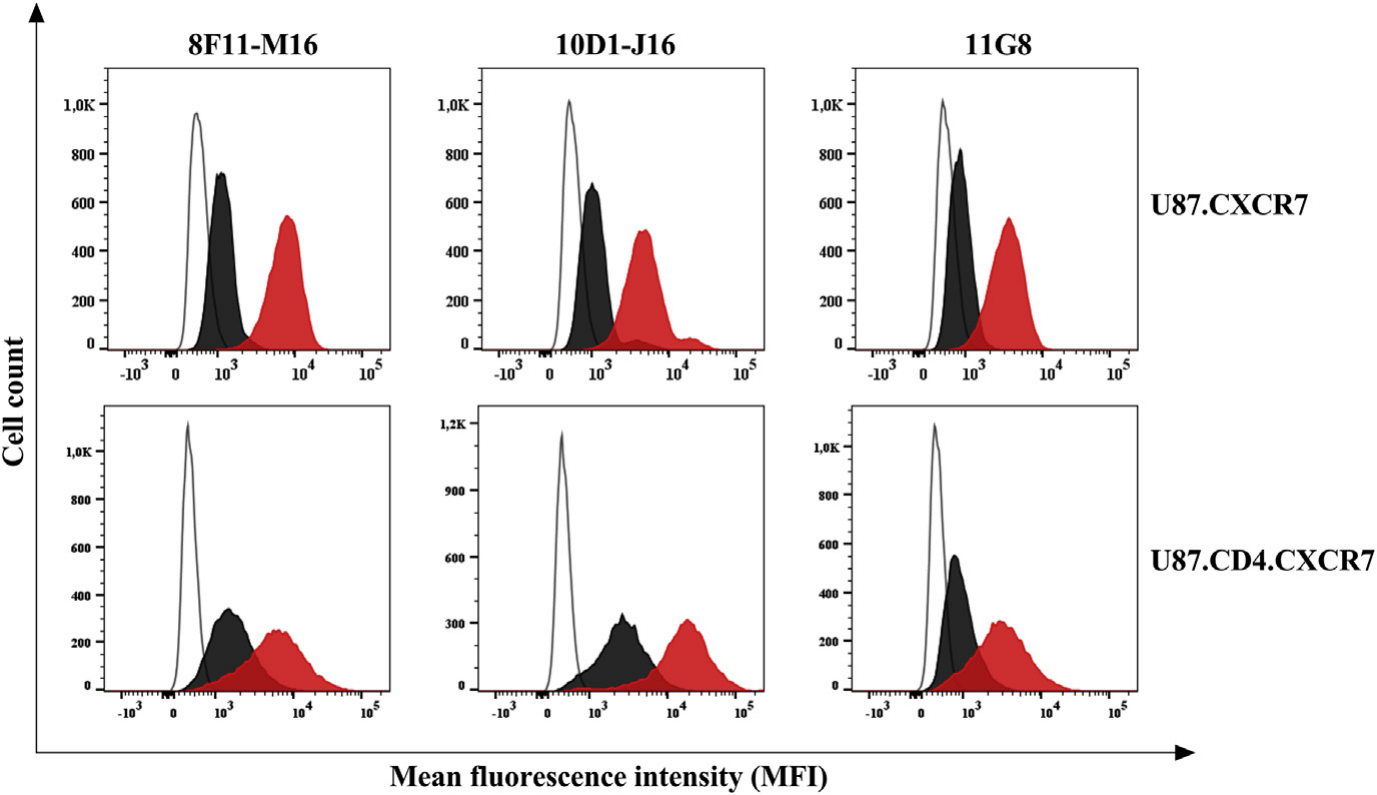
Anti-CXCR7 mAb clone specificity as determined on U87 cells using flow cytometry. Histogram plots of anti-CXCR7 mAb staining are shown to check for clone selectivity in competition experiments between unlabeled and labeled anti-CXCR7 antibodies (black) from the same antibody clone. In each histogram plot, also the mAb clone staining without preincubation with the unlabeled mAb was shown (red). All antibody staining conditions were compared to an unstained control sample (empty histogram). A representative staining was shown from one out of three independent experiments.

After validation of the CXCR7-transfected cell lines, the specificity of the unlabeled anti-CXCR7 mAb clones 8F11-M16, 10D1-J16 and 11G8 was verified (Fig. 3). Here, we checked the specificity of the CXCR7-targeting antibodies via a competition experiment between the unlabeled and labeled form of the same mAb clone. All unlabeled mAbs could efficiently block the binding, and thus receptor staining, of their fluorescently-labeled mAb clone counterpart. Therefore, we are confident that the mAb clones used for cellular receptor staining have the same binding specificity as compared to the clones used for blocking the CXCR7-mediated HIV entry process.

In conclusion, two CXCR7-positive cell lines were created with relatively high receptor expression levels. These cell lines are therefore well-suited to study HIV entry processes via the CXCR7 coreceptor. Secondly, the unlabeled CXCR7-targeting mAb clones that were used as blockers in our CXCR7-dependent HIV entry study were proven specific for CXCR7, as shown by antibody competition experiments with the equivalent labeled antibody.

### 3.2 CXCR7-positive U87 cells can be efficiently infected by HIV-1 and HIV-2 strains

**Fig. 4:**
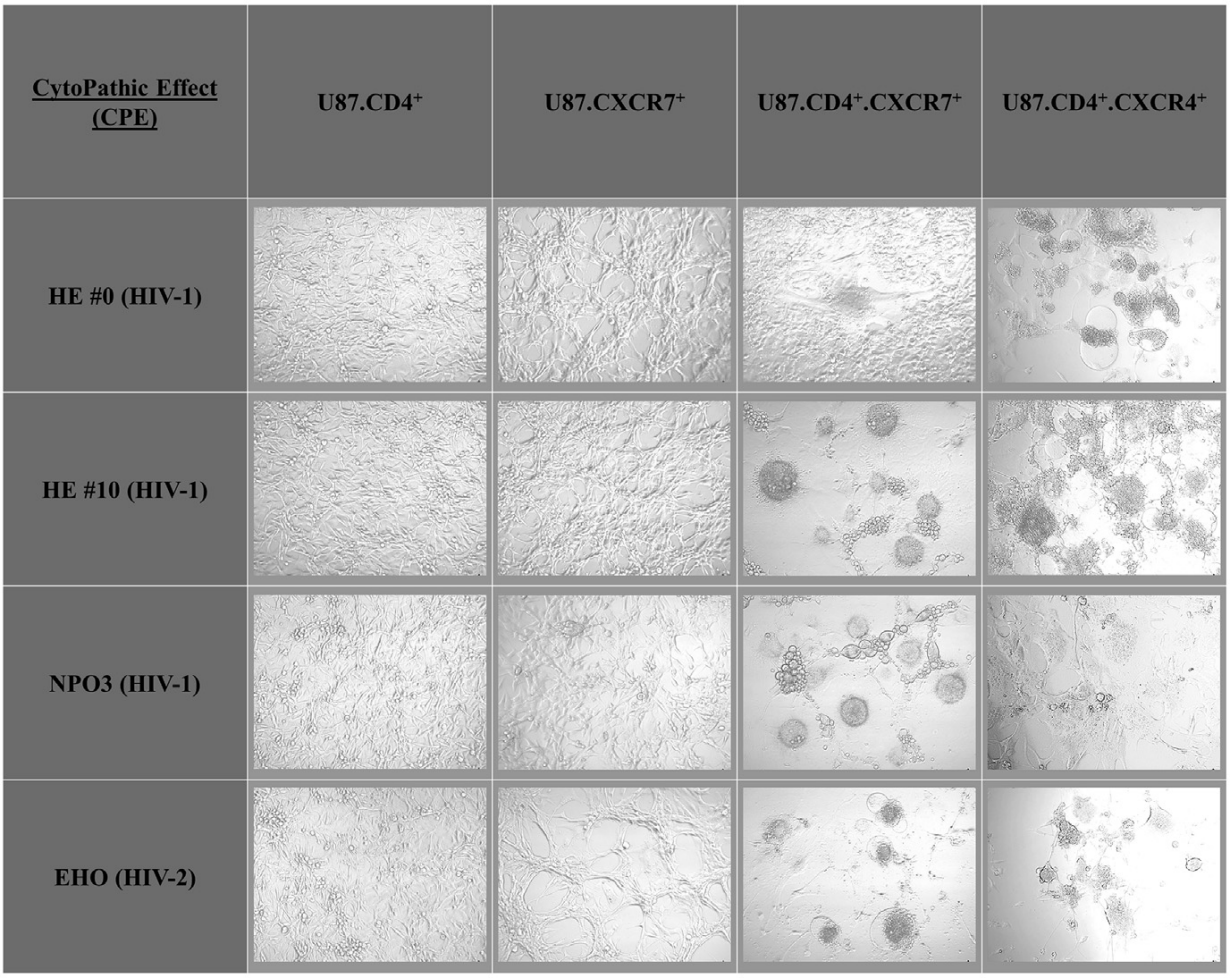
Microscopic evaluation of HIV infection based on virus-induced cytopathic effect (CPE). A representative staining was shown from one out of three independent experiments. Briefly, U87 cells were infected with HIV-1 HE #0, HIV-1 HE #10, HIV-1 NPO3 or HIV-2 EHO (50,000 pg of p24/p27 stock solution) and incubated at 37 °C with 5% CO_2_ for 5 days. Thereafter, pictures were captured on a Zeis Primovert inverted microscope equipped with an Axiocam ERc 5s camera.

An overview of cytopathic effect (CPE) snapshots is shown for HIV strains able to infect human host cells using CXCR7, in addition to CXCR4, as a coreceptor (Fig. 4). From an in-house HIV infection screen, in which a representative array of previously characterized CXCR4-using HIV-1 and HIV-2 isolates (n = 30; using 100,000 pg dosing of each viral strain p24 stock solution) were tested on the U87.CD4.CXCR7 cell line, the HIV-1 NPO3 and HIV-2 EHO clinical isolates were picked up as potent CXCR7-using HIV strains (Supplementary File 1). Infection of CD4 and CXCR7 double positive U87 cells with HIV strains NPO3 and EHO was efficient. In addition, the HIV-1 HE #0 laboratory strain, which only caused a weak CPE on U87.CD4.CXCR7 cells, was continuously passaged on these cells to generate the HIV-1 HE #10 strain that caused pronounced CPE after a five day infection period. The infection rate resulting from the HIV-1 HE #10 strain was eventually comparable to the one seen for the clinical isolates. On the contrary, the viral strains HIV-1 HE #0/#10, HIV-1 NPO3 and HIV-2 EHO were unable to infect single positive U87 cells (U87.CD4 and U87.CXCR7). Thus, based on CPE imaging data, both CD4 and CXCR7 are necessary for efficient HIV infection and replication in U87 cells.

**Fig. 5:**
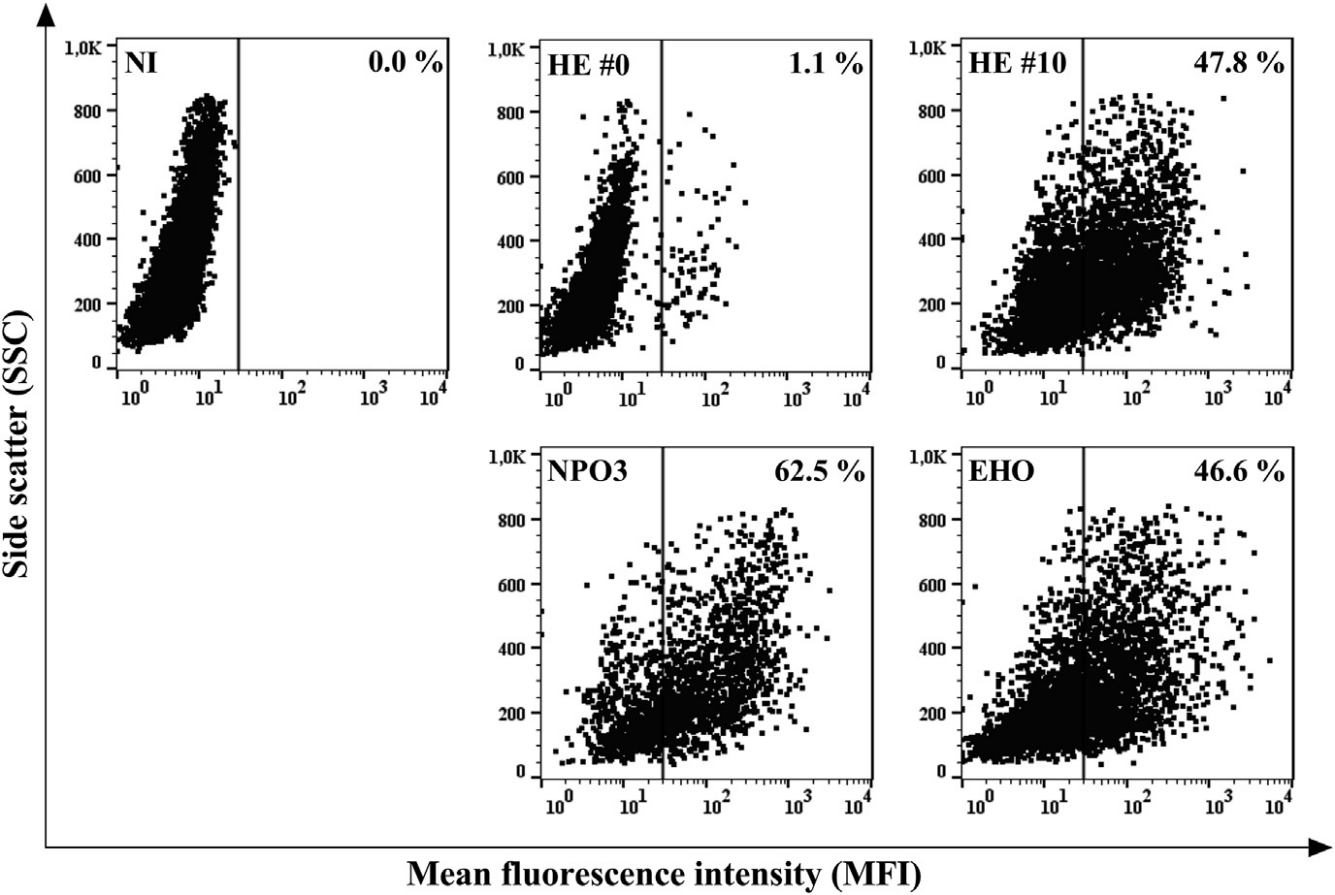
Infection rate of HIV-1 strains HE #0, HE #10 and NPO3, and HIV-2 strain EHO in U87.CD4.CXCR7 cells. Quantification of the % infection resulting from incubation of U87.CD4.CXCR7 cells with HIV-1 HE #0 (100,000 pg), HE #10 (5,000 pg), NPO3 (20,000 pg) and HIV-2 EHO (5,000 pg) was performed via measurement of the HIV p24/p27 capsid protein transcribed by the HIV replication machinery using an intracellular mAb staining against p24/p27. The data were obtained using flow cytometry (each dot plot represents % of infected cells for a given HIV strain). A representative staining was shown from one out of three independent experiments, all resulting in comparable infection rates for the given HIV strains. NI: non-infected.

To confirm the infectious capacity of the viral strains, a flow cytometry-based intracellular antibody staining with a PE-labeled anti-p24/p27 mAb (clone KC57) was performed. This allows to quantify the amount of the p24 (HIV-1) or p27 (HIV-2) capsid proteins transcribed within the U87 host cells by the HIV replication machinery. Relative percentages of intracellular HIV-1 p24 or HIV-2 p27 capsid protein levels of the respective HIV-1 or HIV-2 virions are shown for U87.CD4.CXCR7 cells infected with the HIV-1 strains HE #0 (100,000 pg of p24 stock), HE #10 (5,000 pg) and NPO3 (20,000 pg), and the HIV-2 strain EHO (5,000 pg) (Fig. 5). Thus, improved CXCR7-usage of the passaged HIV-1 HE #10 strain versus HIV-1 HE #0 was confirmed, in concordance with the CPE images (Fig. 4), with an increased infectivity up to 47.8%. HIV-1 NPO3 and HIV-2 EHO also showed very efficient infectivity compared to the non-infected (NI) sample when measuring p24/p27 antigen (Ag) via intracellular protein staining, resulting into an infection rate of 62.5% and 46.6%, respectively (Fig. 5).

HIV-1 and HIV-2 strains typically infect human host cells via initial attachment of the gp120 viral envelope protein to the main HIV entry receptor CD4. Subsequently, binding sites open up on the gp120 protein surface that facilitate interaction with a coreceptor, predominantly CCR5 or CXCR4 [4]. However, this study provides clear evidence of HIV-1 as well as HIV-2 strains being able to infect U87.CD4 cells lacking both CCR5 and CXCR4, but expressing the chemokine receptor CXCR7.

In conclusion, Figs. 4 and 5 show the increased infection rate of the passaged HE #10 strain in comparison to the original HE #0 strain in U87 cells co-expressing CXCR7 and CD4. Furthermore, both figures clearly show the efficiency of the clinical isolates HIV-1 NPO3 and HIV-2 EHO to infect U87.CD4.CXCR7 cells. Our data suggest that the different HIV strains (HE #10, NPO3 and EHO), together with the U87.CD4.CXCR7 cell line, can serve as a suitable cellular model to evaluate potential antivirals targeting the CXCR7 coreceptor.

### 3.3 CXCR7-binding mAbs and small molecules as well as the natural CXCR7 chemokine ligands block X7 HIV-1 and HIV-2 entry and replication

In the next set of experiments, anti-CXCR7 mAbs, CXCR7-targeting small molecules and the natural CXCR7 chemokine ligands were evaluated as candidate inhibitors in the defense against HIV-1 or HIV-2 infection via coreceptor CXCR7. We used U87.CD4.CXCR7 cells for infection and IC_50_ (half minimal (50%) inhibitory concentration (IC) of an inhibitor) calculation was based on the detection of HIV-1 p24 or HIV-2 p27 Ag quantity in the supernatant of infected cell culture using Ag-specific ELISA assays.

The CXCR7-specific mAbs appeared very efficient in inhibiting HIV infection with HIV-1 HE #10 (5,000 pg), HIV-1 NPO3 (20,000 pg) and HIV-2 EHO (5,000 pg) with IC_50_ values ranging between mid ng ml^−1^ to lower μg ml^−1^ values. When comparing anti-CXCR7 mAb clones 8F11-M16, 10D1-J16 and 11G8, the most potent CXCR7 inhibitor was the 10D1-J16 clone, followed closely by 8F11-M16. The 11G8 clone was the least efficient blocker of CXCR7-mediated infection. These observations imply that interaction sites of the 10D1-J16 clone with CXCR7 strongly coincide with the HIV gp120-CXCR7 interaction pattern. In addition, HIV-1 HE #10 was most susceptible to treatment with anti-CXCR7 mAbs (Table 1). This could be attributed to the continuous passaging, and thereby the forced adaptation of HIV-1 HE to U87.CD4.CXCR7 cells. As a consequence, the virus enters the host cells via the most evident entry mechanism but thereby possibly also the easiest gateway for entry inhibition.

**Table 1:** Anti-HIV activity profile of CXCR7 inhibitors on U87.CD4.CXCR7 cells.

HIV p24/p27 Antigen ELISA		Viral strains∗		
		HE #10 (HIV-1)	NPO3 (HIV-1)	EHO (HIV-2)
CXCR7 inhibitors	**Monoclonal antibody**†			
	8F11-M16	0.39 ± 0.04	2.52 ± 1.36	1.35 ± 0.28
	10D1-J16	0.31 ± 0.10	1.28 ± 0.71	0.64 ± 0.08
	11G8	2.64 ± 0.82	3.16 ± 1.48	4.81 ± 1.04
	**Small molecule/peptide**‡			
	CCX771	49.27 ± 18.44	36.78 ± 15.63	>5000
	VUF11207	72.45 ± 25.57	>5000	>5000
	TC 14012	>5000	>5000	>5000
	**Chemokine**‡			
	CXCL11	32.07 ± 6.07	74.07 ± 8.87	25.66 ± 8.11
	CXCL12	231.84 ± 58.78	230.71 ± 29.25	470.26 ± 90.01

∗ Virus input was as follows: 5,000 pg of HIV-1 HE #10, 20,000 pg of HIV-1 NPO3 and 5,000 pg of HIV-2 EHO.

† IC_50_ in μg ml^−1^ required to inhibit viral p24 Ag (for HIV-1) or p27 Ag (for HIV-2) production by 50% in U87.CD4.CXCR7 cells. Mean IC_50_ values with SEM from at least three independent infection experiments are shown.

‡ IC_50_ in nM required to inhibit viral p24 Ag (for HIV-1) or p27 Ag (for HIV-2) production by 50% in U87.CD4.CXCR7 cells. Mean IC_50_ values with SEM from at least three independent infection experiments are shown.

The anti-HIV activity of the CXCR7-targeting small molecule inhibitors CCX771 and VUF11207 was dependent on the viral strain used for infection. CCX771 was only capable of blocking HIV-1 infections with IC_50_ values of ∼50 nM for both HIV-1 HE #10 as well as HIV-1 NPO3 (Table 1). This small chemical compound could not block HIV-2 EHO strain infection when using compound concentrations up to 5000 nM. The activity of VUF11207 was even narrower. For this small molecule, blocking of the HIV-1 HE #10 strain with an IC_50_ value of ∼70 nM was the only observed activity as no IC_50_ value could be calculated for HIV-1 NPO3 and HIV-2 EHO when tested up to 5000 nM. The small cyclic peptide and reported CXCR7 agonist ligand TC14012 had no anti-HIV activity at the tested concentrations (up to 5000 nM) against any of the three X7 HIV strains included in this study.

Finally, the natural CXCR7 ligands CXCL11 and CXCL12 were tested as inhibitors of CXCR7-mediated HIV entry and replication. The IC_50_ values of CXCL11 were in the lower nanomolar range, going from ∼30 nM for HIV-1 HE #10 and HIV-2 EHO inhibition to ∼75 nM for blocking of HIV-1 NPO3 viral entry (Table 1). In comparison, the IC_50_ values of CXCL12 were generally higher with ∼230 nM for HIV-1 HE #10 and HIV-1 NPO3, and ∼470 nM for HIV-2 EHO. According to recent findings from a mutational analysis in Benredjem et al., (2016), there is no involvement of N-terminal CXCR7 interaction residues for CXCL12 binding to the receptor extracellular surface. CXCL11 on the other hand, relies on both N-terminal and extracellular loop contacts for binding to CXCR7 [35]. Therefore, the improved activity profile of CXCL11 compared to CXCL12 in the context of HIV gp120-CXCR7 blocking can be explained by the increased occupancy of the CXCR7 chemokine receptor by CXCL11.

In general, small molecules tend to reside within a specified binding pocket on the receptor's extracellular surface or bind in pockets shaped by neighboring extracellular loops of the chemokine receptor [24, 36, 37, 38]. They can usually only span small, delineated regions on the receptor surface for potential interference with HIV gp120-CXCR7 binding. Thereby the lack of CXCR7-targeting small molecule inhibition of HIV-2 EHO strain infection could be explained, because this virus presumably makes use of a different binding site on the CXCR7 cellular surface to enter its host cells. We suggest that anti-CXCR7 mAbs are by definition broader neutralizing agents, because of their larger molecular size (∼150 kDa), by which they can cover more ground as it comes to extracellular receptor surface. In addition, chemokines -with a molecular weight of approximately 8 kDa- are larger than their small molecule counterparts and therefore also occupy the chemokine receptor CXCR7 to a greater extent [35]. Furthermore, the chemokine ligands CXCL11 and CXCL12 also internalize the CXCR7 receptor, so HIV particles are unable to enter the host cells due to absence of the receptor on the cellular surface [20].

### 3.4 Rapid virion uptake of HIV-1 HE #10 strain in U87.CD4.CXCR7 cells can be blocked by CXCR7-specific inhibitors

**Fig. 6:**
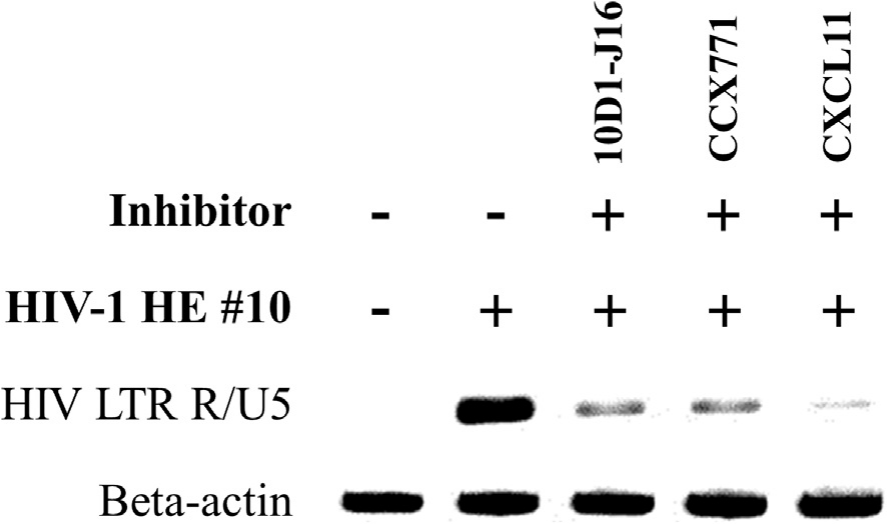
DNA agarose gel showing HIV entry PCR on HIV LTR R/U5 and beta-actin DNA isolated from HIV-1 HE #10 infected U87.CD4.CXCR7 cells. The effect of the CXCR7 inhibitors is shown on U87.CD4.CXCR7 cells infected with 5,000 pg of HIV-1 HE #10. In brief, CD4/CXCR7-positive U87 cells were left untreated or were initially preincubated with the CXCR7 inhibitors 10D1-J16 mAb (10 μg ml^−1^), CCX771 (5 μM) and CXCL11 (1 μM). Thereafter, all experimental conditions, except for the non-infected control, were infected with 5,000 pg HIV-1 HE #10. After a 2 h incubation period at 37 °C, 5% CO_2_ the cells in each well of an experimental condition were lysed and total DNA was isolated. Finally, an HIV-1 specific PCR was performed on each isolated total DNA sample to check for the amount of proviral DNA. The PCR-amplified proviral DNA from the isolated total DNA samples was eventually run and visualized on a 2.2% agarose gel. A full, non-adjusted image of the DNA agarose gel is provided in the Supplementary content (Supplementary File 2).

Elucidation of early HIV-1 entry events into human host cells was done using a PCR-based HIV-1 entry method. Here we used HIV-1-specific DNA as template for HIV infection rate quantification in contrast to the HIV-1 p24 or HIV-2 p27 Ag from supernatant samples used in the p24/p27 ELISA method. Essentially, a semi-quantitative HIV-1 LTR R/U5-specific PCR was performed on total DNA isolated from U87.CD4.CXCR7 cells at 2 h after infection with the X7 HIV-1 HE #10 strain. The synthesis of the LTR R/U5 DNA transcript, also called strong-stop DNA, is the first step of reverse transcription and is completed early in the infection process, shortly after viral entry and uncoating. Total DNA was isolated from U87.CD4.CXCR7 cells that were left untreated or were pretreated for 15 min with the CXCR7 inhibitors 10D1-J16, CCX771 or CXCL11 at the highest dosing in concordance with the p24 ELISA method. A DNA agarose gel was loaded with PCR-amplified HIV-1-specific early transcribed DNA from total DNA samples isolated from uninfected and infected (5,000 pg virus stock) U87.CD4.CXCR7 cells (Fig. 6). Preincubation of U87.CD4.CXCR7 cells with a 10 μg ml^−1^ dosing of the anti-CXCR7 mAb clone 10D1-J16, the CCX771 small molecule CXCR7 antagonist at 5 μM or the CXCR7 ligand CXCL11 at 1 μM resulted in a marked decline in the amount of HIV-1 specific LTR R/U5 DNA. With this, we confirmed the anti-viral potency of these CXCR7-targeting inhibitors with the highest affinity for the HIV coreceptor CXCR7. In addition, this emphasized the efficacy of our HIV-1 entry PCR assay for studying the early infection events with the X7 HIV-1 HE #10 strain.

### 3.5 Blocking of HIV-1 HE #10 strain infection and replication in U87.CD4.CXCR7 cells as determined via quantitative PCR

The efficiency of virus infection and replication in the U87.CD4.CXCR7 cell line was evaluated using a highly sensitive quantitative PCR (qPCR) method for absolute quantification of HIV copy numbers based on the standard curve method. Infection of U87.CD4.CXCR7 cells with 5,000 pg and 10,000 pg X7 HIV-1 HE #10 strain resulted in a dose-dependent infectivity rate with HIV copy numbers of ∼1,800 and ∼5,000 copies, respectively (Table 2).

**Table 2:** Evaluation of CXCR7 inhibitors interfering with HIV entry/replication using qPCR.

Virus input	CXCR7 inhibitor∗	Viral copy number† ± SEM	*% Inhibition*‡*± SEM*
Non-infected	–	0.00 ± 0.00	–
HIV-1 HE #10 5,000 pg	–	1767.97 ± 753.01	–
	10D1-J16	11.56 ± 0.95	*99.23 ± 0.28*
	CCX771	8.38 ± 0.82	*99.40 ± 0.30*
	CXCL11	0.00 ± 0.00	*100.00 ± 0.00*
HIV-1 HE #10 10,000 pg	–	4968.16 ± 714.36	–
	10D1-J16	16.84 ± 5.43	*99.67 ± 0.06*
	CCX771	15.91 ± 1.85	*99.68 ± 0.01*
	CXCL11	14.65 ± 1.91	*99.71 ± 0.00*

∗ The three most efficient CXCR7 inhibitors 10D1-J16, CCX771 and CXCL11 were dosed at 10 μg ml^−1^, 5 μM and 1 μM, respectively, corresponding to the maximal dosing used for the p24 ELISA assay.

† Viral copy numbers were derived from a standard curve created by serial dilution of known amounts of HIV-1 LTR R/U5 cDNA containing plasmid vector. All DNA samples were analyzed in triplicate within each qPCR experiment, and mean viral copy numbers with SEM from two biological replicates per experimental condition are shown.

‡ Mean values of % Inhibition with SEM are shown from two biological replicates analyzed in triplicate within each qPCR experiment.

In addition, the viral copy numbers detected in HIV-1 HE #10 strain infected (5,000 pg versus 10,000 pg) U87.CD4.CXCR7 cells that were pretreated with CXCR7 ligands 10D1-J16 (10 μg ml^−1^), CCX771 (5 μM) or CXCL11 (1 μM) ranged from near zero to ∼17 copies. Consequently, a maximal inhibitory efficiency was obtained with these compounds, when compared to the infected, untreated sample for each virus input. The data from these qPCR experiments confirm the potencies of the evaluated CXCR7 inhibitors that show similar inhibitory efficacy when compared to the p24 ELISA experiments. Taken together, the qPCR results along with the results from the semi-quantitative HIV entry experiments underscore the role for CXCR7 inhibitors as HIV entry blockers, but also for interference with the subsequent HIV replication process.

## 4 Conclusions

In this study, CXCR7-mediated viral entry of HIV-1 and HIV-2 strains was demonstrated experimentally using a novel *in vitro* cellular infection model, in which CXCR7 was the sole chemokine receptor overexpressed on the surface of human U87 glioblastoma cells along with CD4, the main HIV receptor. The anti-CXCR7 mAb clones 8F11-M16, 10D1-J16 and 11G8 consistently and efficiently inhibited CXCR7-mediated HIV entry at the host cell surface, which confirmed that the viral entry genuinely occurred via CXCR7. The natural CXCR7 chemokine ligands CXCL11 and CXCL12 also efficiently blocked HIV entry via CXCR7. The antiviral potency of these chemokines might occur via an inhibition mechanism that combines multi-site extracellular surface blocking and removal of CXCR7 from the cell surface via receptor endocytosis. The less consistent inhibition profile of the tested small molecule CXCR7 ligands CCX771, VUF11207 and TC14012 could instead be attributed to their smaller molecular size and deviant amino acid interactions with CXCR7 compared to gp120-CXCR7 interaction sites. Nevertheless, the three classes of gp120-CXCR7 interfering ligands identified within this study make up useful starting points for counteracting HIV strains and clinical isolates that have adapted to CXCR7 coreceptor use during early infection.

Of interest, the CXCR7 inhibitor CCX771 was recently described to interfere with CXCL12-directed migration of CXCR7-positive CD14^+^ CD16^+^ monocytes of HIV-infected people into the CNS, which can result in a neurodegenerative disease termed NeuroAIDS [39]. Under normal physiological conditions, double positive CD14^+^ CD16^+^ monocytes constitute only 10–15% of the total monocyte population. However, an increase in these nonclassic monocytes is associated with several pathologies, including HIV infection [40, 41]. Aside from the role of CXCR7 in CXCL12-guided cell migration across the blood brain barrier (BBB), the higher CXCR7 cell surface expression on the HIV-susceptible CD14^+^ CD16^+^ monocytes might increase the likelihood of CXCR7 coreceptor use in subsequent infection cycles of CD14^+^ CD16^+^ monocytes. A major challenge, however, lies in assessing the role of CXCR7 as coreceptor in human monocyte (sub)populations, since different monocyte populations carry varying subsets of a whole range of candidate HIV coreceptors on their cell surface [42, 43]. Besides monocytes, CXCR7 was also expressed on the cell surface of other human leukocytes, among which subsets of B cells and T cells. For instance, CXCR7-expressing B cells were elevated in HIV-positive individuals [44, 45]. In addition, increased CXCR7 expression on CD4^+^ T cells was associated with immune dysregulation in children with autism [46]. The latter observations in defined B and T cell subpopulations could point to possible CXCR7 coreceptor use in case of immune imbalance.

Finally, the emergence of different reports on cross-talk between CXCR4 and CXCR7 with regard to CXCL12/CXCR4/CXCR7 downstream signaling [47, 48, 49, 50, 51, 52, 53] within the last decade strengthens the presumption that CXCR7 is used by more HIV strains than we can currently comprehend. In conclusion, the flexibility of HIV particles to adapt to a broader range of coreceptors or to an alternative coreceptor use could be strongly underestimated when keeping in mind the intimate CXCR4/CXCR7 relationship and the currently increasing circulation of CXCR4-using viruses in South American and Asian countries, which implies a coinciding increase in CXCR7-using HIV strains [8, 9].

## Declarations

### Author contribution statement

Thomas D'huys: Conceived and designed the experiments; Performed the experiments; Analyzed and interpreted the data; Wrote the paper.

Sandra Claes: Performed the experiments.

Tom Van Loy: Analyzed and interpreted the data; Wrote the paper.

Dominique Schols: Conceived and designed the experiments; Analyzed and interpreted the data; Wrote the paper.

### Funding statement

This work was supported by the KU Leuven (https://www.kuleuven.be, grant no. PF/10/018), the Fonds voor Wetenschappelijk Onderzoek (http://www.fwo.be, grant no. G.485.08) and the Fondation Dormeur Vaduz.

### Competing interest statement

The authors declare no conflict of interest.

### Additional information

No additional information is available for this paper.
