# Supplementary material for: CXCR7/ACKR3-targeting ligands interfere with X7 HIV-1 and HIV-2 entry and replication in human host cells
Source: Heliyon. 2018 Mar 1;4(3):e00557. doi: 10.1016/j.heliyon.2018.e00557 (PMC5857896; doi:10.1016/j.heliyon.2018.e00557)
Supplement: Supplementary File 1 [file mmc1.docx]

**Supplementary File 1. List of CXCR4-using HIV clinical isolates tested for infection of U87.CD4.CXCR7 cells.**

| Clinical isolate (CXCR4-using) | HIV-1 or HIV-2 | Subtype | CPE |
| --- | --- | --- | --- |
| **I-2496** | HIV-1 | A | - |
| **UG273** | HIV-1 | A | - |
| **93/US/141** | HIV-1 | B | - |
| **US4** | HIV-1 | B | - |
| **BK132** | HIV-1 | B | - |
| **I-2516** | HIV-1 | C | - |
| **ETH2220** | HIV-1 | C | - |
| **ZAM18** | HIV-1 | C | - |
| **DJ259** | HIV-1 | C | - |
| **SM145** | HIV-1 | C | - |
| **SE365** | HIV-1 | D | - |
| **UG270** | HIV-1 | D | - |
| **BZ162** | HIV-1 | F | - |
| **BZ-163** | HIV-1 | F | -/+ |
| **BCI-RO7** | HIV-1 | F | -/+ |
| **BCF-DIOUM** | HIV-1 | G | - |
| **RU-570** | HIV-1 | G | - |
| **BCF-KITA** | HIV-1 | H | - |
| **BCF06** | HIV-1 | Group O | -/+ |
| **I-2478B** | HIV-1 | Group O | - |
| **CM240** | HIV-1 | CRF01 AE | - |
| **ID12** | HIV-1 | CRF01 AE | - |
| **NPO3** | HIV-1 | CRF01 AE | + |
| **ID17** | HIV-1 | CRF01 AE | - |
| **NP1465** | HIV-1 | CRF01 AE | - |
| **CM243** | HIV-1 | CRF01 AE | - |
| **CI42368** | HIV-1 | CRF01 AE | - |
| **POC 44951** | HIV-1 | CRF02 AG | - |
| **EHO** | HIV-2 | / | + |
| **BV5061W** | HIV-2 | / | - |

* All clinical isolates were provided by Dr. J. Lathey (then at BBI Biotech Research Laboratories, Gaithersburg, MD, USA), except for HIV-1 isolate RU-570 provided by the Institute of Tropical Medicine (Antwerp, Belgium) and the clinical isolate HIV-2 EHO. The HIV infection screen was set up with a virus dosing at 100,000 pg of p24 stock solution.
