# Supplementary material for: CXCR7/ACKR3-targeting ligands interfere with X7 HIV-1 and HIV-2 entry and replication in human host cells
Source: Heliyon. 2018 Mar 1;4(3):e00557. doi: 10.1016/j.heliyon.2018.e00557 (PMC5857896; doi:10.1016/j.heliyon.2018.e00557)

**Supplementary File 2. Full, non-adjusted image of DNA agarose gel from Fig. 6 showing HIV entry PCR on HIV LTR R/U5 and beta-actin DNA isolated from HIV-1 HE #10 infected U87.CD4.CXCR7 cells.**


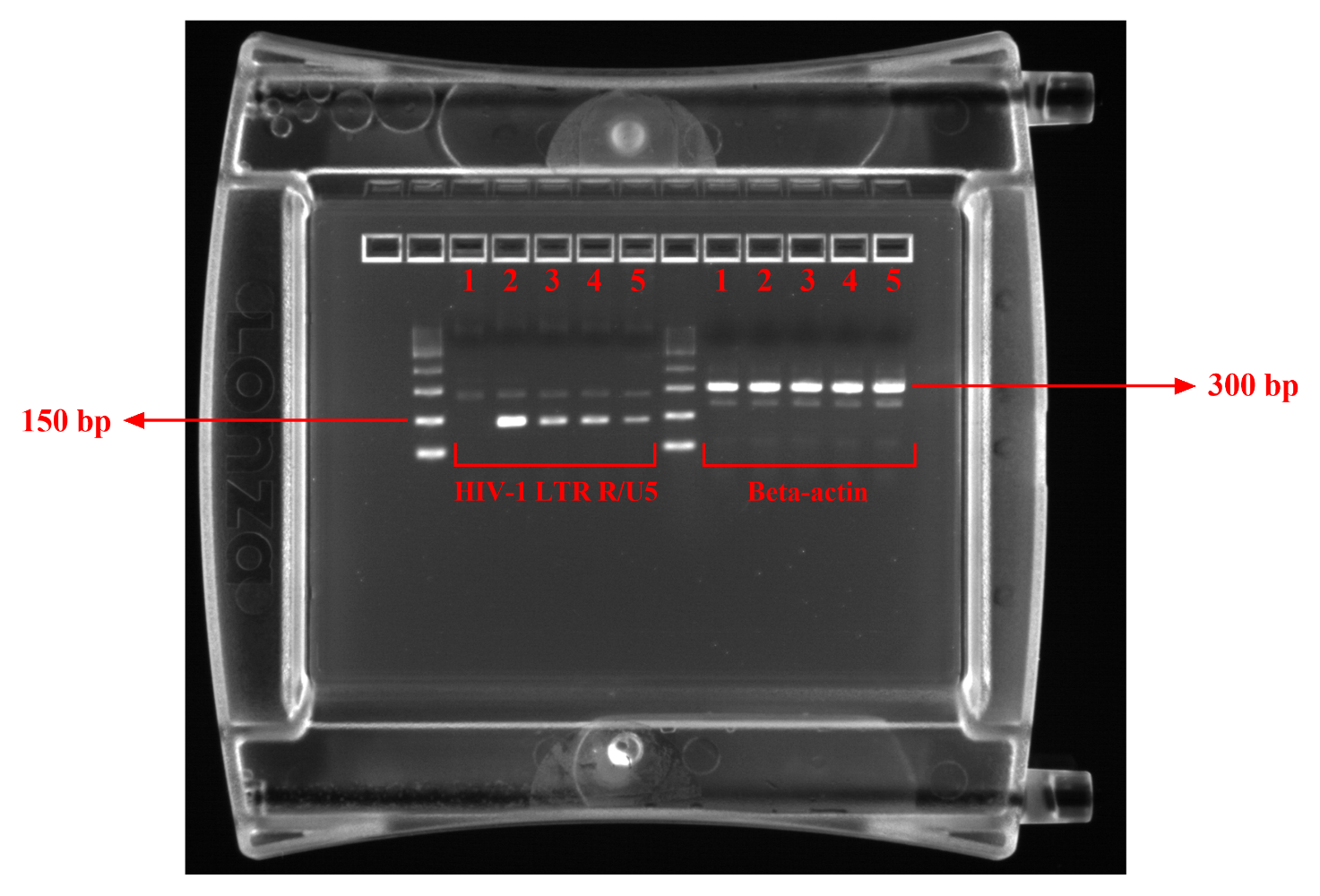

Supplement: Supplementary File 2 [file mmc2.docx]
